# Combinatorial Maps, a New Framework to Model Agroforestry Systems

**DOI:** 10.34133/plantphenomics.0120

**Published:** 2023-12-15

**Authors:** Laëtitia Lemiere, Marc Jaeger, Marie Gosme, Gérard Subsol

**Affiliations:** ^1^ ABSys, Univ Montpellier, CIHEAM-IAMM, CIRAD, INRAE, Institut Agro, Montpellier, France.; ^2^ CIRAD, UMR AMAP, F-34398 Montpellier, France.; ^3^ AMAP, Univ Montpellier, CIRAD, CNRS, INRAE, IRD, Montpellier, France.; ^4^ Research-Team ICAR, LIRMM, Univ Montpellier, CNRS, Montpellier, France.

## Abstract

Agroforestry systems are complex due to the diverse interactions between their elements, and they develop over several decades. Existing numerical models focus either on the structure or on the functions of agroforestry systems. However, both of these aspects are necessary, as function influences structure and vice versa. Here, we present a representation of agroforestry systems based on combinatorial maps (which are a type of multidimensional graphs), that allows conceptualizing the structure–function relationship at the agroecosystem scale. We show that such a model can represent the structure of agroforestry systems at multiple scales and its evolution through time. We propose an implementation of this framework, coded in Python, which is available on GitHub. In the future, this framework could be coupled with knowledge based or with biophysical simulation models to predict the production of ecosystem services. The code can also be integrated into visualization tools. Combinatorial maps seem promising to provide a unifying and generic description of agroforestry systems, including their structure, functions, and dynamics, with the possibility to translate to and from other representations.

## Introduction

### Agroforestry

Agroforestry systems (AFS) are composed of a mixture of trees, crops, and/or animals [1] providing many ecosystem services beneficial to humans [2]. These systems are complex due to the high level of biodiversity that they contain (both planted and spontaneous), and the interactions between the different species. Since interactions between species act locally, AFS are characterized by a high level of spatial heterogeneity, and the spatial arrangement of the components of the system is a crucial driver of its functioning [3]. Therefore, the spatial design of these systems determines the production of ecosystem services [4]. Furthermore, AFS develop over a long period due to the slow growth of trees. AFS evolve through time due to

• internal dynamic processes (tree growth is an example, which results in increased shade)
• farmers’ management, which can have an impact on the system’s structure (for instance tree thinning).

Some choices made at plantation time (such as tree row distance and orientation) have consequences over the whole lifetime of the system. Conversely, some aspects of the management of these systems must be adaptive over time. In fact, as the trees grow and the shade becomes more important, the cultivated species and management change.

### Models: From crop models to FSPM

Due to the diversity of possible combinations between tree, crop, animal species, and the associated management, and to the long development time of AFS, experimental evidence is scarce to understand the functioning, measure the performance and optimize the design of AFS. Simulation models would be of great help in this respect. Plant modeling has a long history, starting in the 60s [5,6] aiming to predict crop yields in relation to environmental conditions. Such models, designed at the crop level (and thus so-called crop models), became mature in the late 90s [7] and have since been used to evaluate cropping systems, including systems based on diversified rotations [8]. However, these models fail to explain within field variability, cannot be employed when secondary growth must be considered (trees), and are not adapted to crop mixtures beyond simple 2-species mixtures [9,10].

In these cases, structural aspects should be considered, downscaling the model at the individual plant level. This can be effective using functional–structural plant models (FSPM), whose development started in the late 90s [11]. FSPM rely on the strong mutual links between the plant architecture (related to structure) and the ecophysiological processes (related to functions and specifically production) [12]. FSPM are often used to model and visualize plants [13] but also to predict the production of ecosystem services, either one by one [14] or several at a time [15]. FSPM can be used as a decision support tool [16] to help farmers make decisions to optimize crop performance. However, FSPM often face the drawback of complexity in their calibration and validation, thus limiting their use in agroecology, which relies on species diversification. Subject to specific assumptions, it is possible to use an approach based on cohorts to overcome this limitation, as was done in the Greenlab model [17], thus bridging the scale from individual plant to crop scale [18]. Nevertheless, they fail to give a conceptual modeling framework for AFS, since traditionally they were not able to address simultaneously different species. Recently, they have been used to optimize mixtures of several plants [19], but these works remain limited, in scope and genericity. Agroforestry models have rarely used a FSPM approach [20]. When they did, they focused on individual plant architecture (for instance to define tree structural plasticity from local environmental conditions [21,22] and not the spatial organization of the system itself.

### Limits of existing models of the structure of AFS

**Fig. 1.**
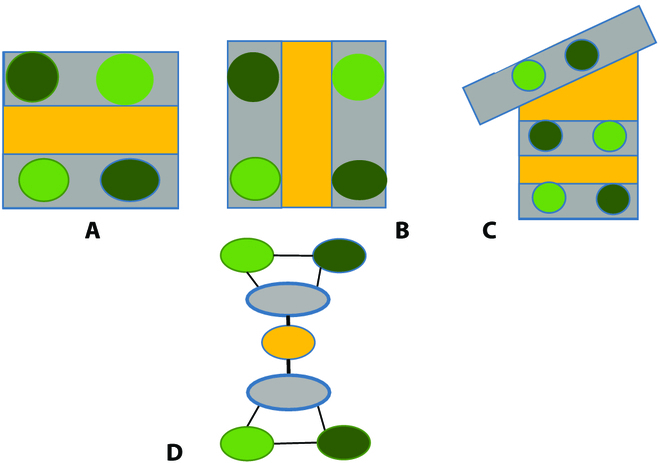
Three different agroforestry plots (A to C) represented by the same graph (D).

Existing agroforestry models (both simulation models and models aiming at visualization) have used a variety of ways to represent the structure of AFS. In the most abstract representations, tree positions are not explicitly represented, but only the topology, i.e., the adjacency relationships between the elements composing the agroforestry system. This results in graph representations of AFS [23]. Graphs are built from nodes, representing the elements composing the system, and directional edges linking them, representing node relations such as adjacency. Graphs offer a structural description of composition and adjacency, as presented in Fig. 1, and can also be mobilized to represent the dynamics of the system. However, contrary to FSPM, they do not emphasize nor take advantage of the strong link between structure and function. An in-between could be to associate interactions between elements (including ecosystem services) to edges between the nodes. Then, simple algorithms can be applied to model the evolution of the plot [24] or the ecosystem services [25]. However, this representation is not sufficient to explicit the spatial arrangement of an agroforestry system. For example, Fig. 1A to C show that 3 different plots may share the underlying graph (Fig. 1D).

One possible solution to this problem is to explicit the distances between the elements of the system and their relative positions, for a representative part of the system, i.e., to identify the Ecosystem Services Spatial Unit. The Ecosystem Services Spatial Unit is the smallest spatial unit encompassing all the interacting species and other functional components that together provide a specified set of ecosystem services in a farming landscape [26]. This is the option chosen in WaNulCas [27] and Hi-sAfe [28]. They simulate the functioning of a simple pattern (3 trees maximum for WaNulCas; any number, but the smallest possible for computational costs, for Hi-sAfe), which can then be reproduced by defining periodic boundary conditions to simulate an infinite space and avoid edge effects. Then, the spatial heterogeneity of AFS is modeled thanks to a discretization of space. WaNulCas operates in 2D, using the distances from the tree locations and the depth in soil, while Hi-sAFe operates in 3D, allowing representation of soil layers as well as distance and orientation to the tree. The advantage of these methods is that they reduce the computation cost: instead of simulating the whole plot, only the pattern is simulated. The disadvantage is that these models cannot represent constraints at the plot level, such as the width of the headlands to allow farm machinery to turn.

Other agroforestry models focus only on the structure and do not have the capacity to represent the functioning. This is the case with EcoAf [29], or RegenWorks [30], which uses a rule-based description. In these cases, the agroforestry system is described by the combination of a pattern (e.g., repetition of lines with a certain orientation and distance between lines, with a succession of trees along the lines) and rules (e.g., “the first line must be at least 20 m from the plot boundary” to allow for machine maneuvers, “no line should be shorter than 10 m” to avoid having trees in the corners of the plot). The pattern and rules can then be applied to any georeferenced plot to generate an instantiation of an agroforestry system, obtaining the geographical coordinates of trees to be used when planting in the field. The advantage of this method is that it takes into account some of the constraints at the plot level, but it does not simulate the links between structure and function. For example, in EcoAF, tree growth is simulated by simple look-up tables that predict the size of a tree as a function of its age, but it does not take into account its environment. Furthermore, rule-based models are not able to represent irregular systems such as scattered trees found in traditional AFS, or even in modern AFS, when tree mortality creates more or less random gaps in the initial pattern after a few years.

To overcome this problem and to represent accurately an agroforestry system, models can use the coordinates of each individual tree, either with local coordinates [31] or with a geographic coordinate system. To date, there are few examples employing formal approaches from the Geographic Information System (GIS) world to agroforestry system design. A formalism can be derived from a dedicated language as proposed by Degenne et al. [32] and can also be implemented as graphs in the generic Ocelet platform [33]. This way of representing AFS is close to field reality, but simulating the functioning of AFS can become very expensive in terms of computational time in the case of large plots. Moreover, models using this representation of space often rely on basic relationships to simulate functions as a linear function between tree size and age as shown in Agroforestryx [34] or ShadeMotion [35].

Table 1 compares these different spatial representations in terms of complexity, spatial, and time criteria. Not surprisingly, there is a trade-off between realism in the spatial representation of agroforestry system and versatility, i.e., the ability to be applied to different plots/AFS with minimal additional effort. There seems to be an opposition between those focusing on the structure of AFS (GIS-based, rule-based) and the ones focusing on the functioning of AFS: realistic representations lack many features that would be desirable for the simulation of agronomic performance and ecosystem service production, such as the representation of interactions between species. Technical performances are also considered such as computation costs. Another lack is the interaction with the outside world: in spatial unit-based representations, the outside world does not exist because the pattern is replicated indefinitely. In rule-based representations, the outside is just represented by constraints linked with the shape of the plot, and in GIS-based representations, the outside world is not explicitly represented or not considered.

**Table 1.** Qualitative comparison of different types of spatial representations of AFS

	Model of AFS			
Comparison criteria	Graph	Spatial unit	Rules-based	GIS
Ability to simulate different plots/AFS with small extra effort	++	++	+	−
Efficiency to reduce computation costs necessary to simulate ecosystem services	++	+	−	−
Ability to simulate local interactions between species	++	+	−	−
Ability to take into account constraints at plot scale	−	−	++	+
Ability to represent understory vegetation strips	−	−	+	−
Ability to represent irregular-shaped AFS	+	−	−	++
Realism of the spatial representation	−	−	+	++
Interaction of the system with outside	+	−	−	−
Ability to project in time	+	+	+	−
Example	[23]	Hi-Safe [28]	EcoAf [29]	[31]

**Table 2.** Combinatorial map’s elements and their corresponding meaning in agroforestry context

Combinatorial map object	Map interpretation	Example (red, Fig. 5B)	Interpretation in dual	Example (blue, Fig. 5B)
Dart	A side of the current element	1: External right border of the full system	A link from the current element to the other	1: Interface between the understory vegetation strip to crop
β1 (permutation)	Pass to next dart: follow the element border	β1(1) = 2 links to lower right external border	Go to next element issued from the current element	β1(1) = 2 : Move from strip-crop link to crop-outside
β2 (involution)	Go to inverse dart to change face: move to neighbor face	β2(1) = 9 links the external right border to the line strip right border	Identify the element is connected from	β2(11) = 12Upper left tree is connected to left line tree strip
Compound operations
OrbitFace	Path through all darts from the same face	(β1 o β1 o β1 o β1)(9) = β1*(9) follows the right line tree strip starting from its right border (9)	(β1 o β1 o β1 o β1)(9) follows the right line tree strip starting from its right border (9)	(β1 o β1 o β1 o β1)(9) follows the right line tree strip starting from its right border (9)
OrbitNode	Intersection	OrbitNode (16) = (β1 o β2)^*^(16) defines the node B from darts 16, 13,11,12,2,3	Area, identifies all relations issuing from an element	Orbit(Crop): relations with top line tree strip, outside and bottom line-tree strip

Finally, AFS and the ecosystem services they provide evolve over the seasons and years. Thus, it is important that the spatial representation of AFS allows representing the evolution of the structure through time, a feature about which the existing representations of AFS are not very good at.

As a summary, the use of graph shows wider benefits from other approaches, and our hypothesis was that the identified drawbacks can be overcome:

• The ability to take into account constraints at plot scale may be solved using a hierarchical multiscale graph definition able to consider the system as a whole in interaction with the outside.
• The ability to represent understory vegetation strip can be assessed using a classical multilayer approach, or even better, using a 3-dimensional graph approach representing systems components as solids and thus offering surfaces adjacency as exchange areas.
• The realism of the representation can be assessed by refining the geometry of the components, not only on the nodes but also on the edges, allowing representations of fluxes and interfaces.

Among the various graph class formalisms, combinatorial maps [36] answer to these requirements.

In this paper, we introduce a new framework, inspired by FSPM approaches, to model AFS. This framework combines the versatility and abstracting power of combinatorial maps, with the spatial realism of coordinate-based representations.

## Materials and Methods

In this section, we first introduce the combinatorial maps concepts, requested to describe the systems, and then introduced the combinatorial map dual, which is of high interest to model exchanges between the system components.

Combinatorial maps [37,38] are a theoretical framework that owes its origin to the modeling of the evolution of surfaces, applied to leaf growth simulation [39] and which has been mainly exploited in computer-aided design, mostly for efficient adaptive meshing. We propose this framework to explore to model AFS. The main motivation for choosing this framework is to rely on strong theoretical background based on graph theory. A graph is composed of nodes linked by directional edges. Its background ensures the possibility to add or delete nodes, which entails changes to the edges and faces, in a clean and robust manner, mechanically impacting the attributes. This is important for AFS’ representation, to represent both the dynamics of ecosystem provision within the year, and the dynamics of the system’s structure across years.

### Combinatorial maps

A combinatorial map of dimension n can be seen as a generalization of a graph defined in n dimensions adapted to model spatial subdivisions. Here, we will work in 2 dimensions.

**Fig. 2.**
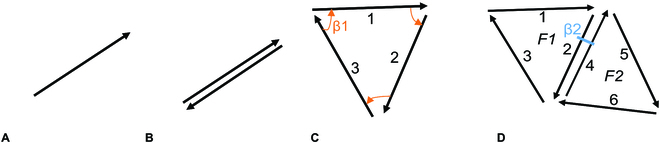
Examples of combinatorial map objects. (A) A dart, (B) an edge, (C) a face, and (D) 2 faces (F1 and F2) with β2, which sets the adjacency between F1 and F2 and allows to move from one face to another.

A combinatorial map of dimension 2 is composed of a limited set of darts (Fig. 2A) and 2 operators defined on the set of darts. The following paragraphs explain in more detail the terms and operations relevant to combinatorial maps.

A dart links 2 nodes. It is oriented. Also, a dart can carry the relationship between the origin of the dart and its end (Fig. 2A).

The nodes, usually connected to spatial points, are defined as a starting (or ending) point of the darts. In our application, a node will represent a plant, a tree, or a tight group of plant. The mathematical definition will be given below because we need to define others elements before.

The faces are the areas surrounded by a closed list of consecutive darts (therefore, dart orientation matters).

The consecutive darts are defined with the permutation operator β1. It finds around the starting node of a given dart the dart that has the smallest angle (given an orientation convention). For example, in Fig. 2C, β1(1) gives 2.

To create a conventional edge that is not oriented (Fig. 2B), we need to have 2 darts linking the same edges but of opposite directions. To do this, we need to define an involution, written β2. It outputs the dart opposite a given dart. Therefore, for any dart *d*, *β2(β2(d)) = d* and in Fig. 2D, *β2(2) = 4* (and* β2(4) = 2*).

A compound sequence of permutation(s) and involution(s) which allows one to traverse the map or a portion of the map from a specific dart *d*, defines an orbit of *d*. Orbits therefore define generic functions as a list of *k* composed operators. Mathematically, an orbit of a given dart *d* can be written as *(βi1 o βi2 o … βik)(d)*, with* ik = 1* *or 2*. For example, in Fig. 2C, the orbit drawing a face from dart 2, is the sequence of repeated *β1* permutation, noted β1*, until reaching dart 2; the sequence is thus 2, *β1(2) = 3*, *β1(3) = 1*,* β1(1) = 2* and its compound function is *(β1 o β1 o β1)*. This specific orbit, applying *β1* until reaching the initial dart *d*, allows us obtaining the face issued from dart *d*; we call it “orbitFace”. This function can then also be used in orbit sequences to visit several faces and in particular the full map. For example, a way to explore the full map drawn in Fig. 2D starting from dart 2 can be the sequence: *orbitFace(2)*, then *β2(2)* and *orbitFace(4)*. It may also be written as *orbitFace(β2(orbitFace(2)))*.

Another useful orbit is the function that allows exploring all darts from a given node. (In fact, in combinatorial map, the node definition raises from this orbit: a node referred from dart *d* is defined by the successive *β1* and *β2 *compound iterations applied on *d*.) We call this function, “orbitNode”, mathematically noted (β1 o β2)*. In Fig. 2D, starting from dart 1,* orbitNode(1)* is a node defined by 1, *β1(1) = 2*, *β2(2) = 4*, *β1(4) = 5*; *β2(5)* is empty.

**Fig. 3.**
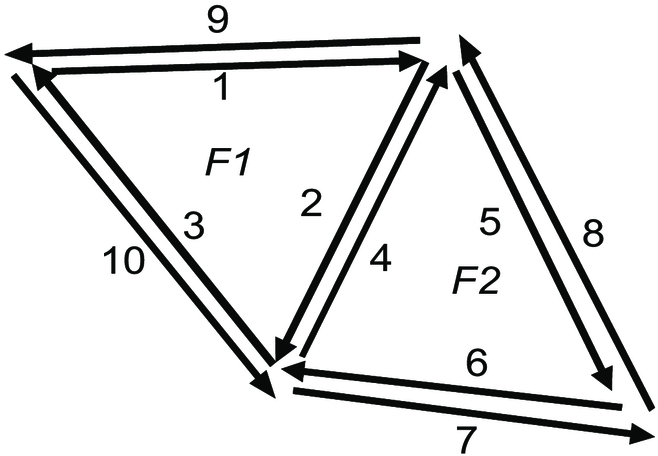
Combinatorial map composed of 2 faces and an external border.

On this example, we see that some dart (such as dart 5) may have no image from the involution: this is the case for each dart lying on the external border of the system. We found it more explicit to constraint the combinatorial map to explicit this external border. Indeed, we consider that the adjacency of an element with the outside of the system is of interest in the case of AFS. On Fig. 2D, this leads to define the outside face introducing 4 new darts: 7, 8, 9, and 10 (see Fig. 3). Mathematically, for each dart *d*, *β2(d)* belongs to the set of darts of the map, and the orbitNode function applies on any dart until reaching it again. With this constraint, orbitFace and orbitNode define a cyclic operator.

Using combinatorial maps allow us to add or delete darts in a formal framework. If we delete an edge to merge the 2 triangle faces from Fig. 2D, all darts must be updated (orientation, update to the next dart) to maintain the map consistency. This possibility is very important for scene design and will be detailed in the discussion section.

### Dual representation

**Fig. 4.**
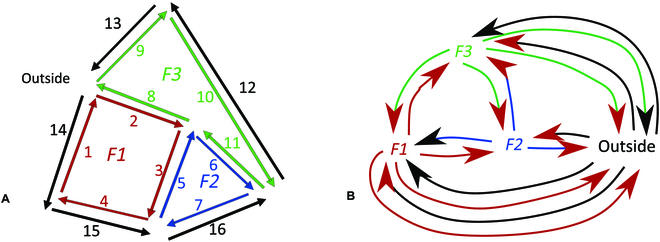
(A) A combinatorial map example and (B) its dual, which is also a combinatorial map.

Graph theory shows that one can construct a dual graph [40] from the original graph called primal. This applies to a combinatorial map and its dual representation, which is also a combinatorial map. It is interesting since it allows us to explicit higher-level adjacency relations automatically. Briefly, to construct the dual, each face becomes a node and each dart links the node corresponding to its face in primal to the node of its *β2*’s face. An example is given in Fig. 4A showing a combinatorial map composed of 3 faces (*F1*, *F2*, and *F3*) and its dual (Fig. 4B). In its dual, *F1*, *F2*, and *F3* become nodes and, for example, edges 3 and 5 between *F1* and *F2* in Fig. 4A become edges connecting nodes *F1* and *F2*. Note that the *β1* and *β2* operators are still the same in the dual.

To explicit the contribution of combinatorial maps, we decline it on an agroforestry context in the following results section and we detail our implementation.

## Results

### Application of combinatorial maps to AFS: A simple example

Starting from a simple example, we illustrate here the underlying concepts and operators of its combinatorial map representation and introduce their respective conceptual meaning in our application on agroforestry system modeling.

**Fig. 5.**
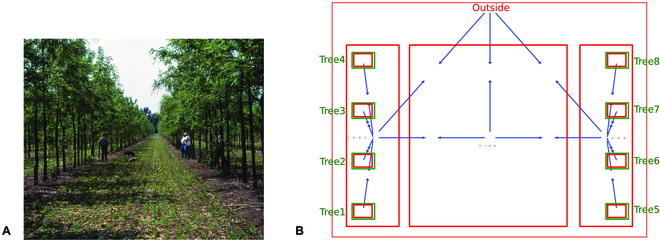
Example of agroforestry plot in combinatorial map. (A) Example of an agroforestry plot with 1 alley of maize crop between 2 lines of trees (credit National Agroforestry Center) (B) A corresponding combinatorial map in red and its corresponding dual in blue, reduced to strips of 4 trees each generated by our application and postprocessed for improved readability.

Let us consider a simple agroforestry system composed of 2 tree lines, each planted on an understory vegetation strip, and 1 crop alley between them, as presented in Fig. 5. Let us also suppose that each tree line is composed of 3 trees. Its combinatorial map representation is shown in Fig. 5B in red. Each area (the 2 strips, trees inside the strips, and crop alley) is modeled by a simple rectangular face. Note that a face (such as a tree line) may contain other faces (trees). This is represented by a hole, a concept natively present in combinatorial map theory and that implies a hierarchical relationship between faces. In a tree line, we represent a tree as a face inside the strip face. At the highest level, the outside face (in red in Fig. 5B) encapsulates the plot composed of 3 faces (2 tree lines and 1 crop alley). An edge is an interface between areas and corresponds to the border either between crop and a tree line or between the plot and the outside of the plot.

In its dual map (the blue arrows in Fig. 5B), the agroforestry areas (the faces, including the outside face) become nodes, and the edges now represent the interactions between areas. Thus, in this dual representation, permutation **β1** can be used to model exchange between different areas. The meaning—the interpretation in our application domain—of the different theoretical elements composing a combinatorial map, its dual, and key operators, are exposed in Table 2, the first 3 lines concerns the combinatorial map conceptual components, dart and operators; while the following lines consider orbits, that is to say compound operators.

### Usefulness of combinatorial maps to represent the functioning of AFS

In complex systems, making sure that a model is consistent (in particular that modifications in one part of the model do not induce undesired consequences on another part) is challenging due to the numerous elements and all their possible interactions. The combinatorial map framework brings a straightforward answer to this question: the list of all aspects that must be considered is simply the list of all cycles in the map and its dual. Indeed, the main interest of using the formalism of combinatorial maps is the proper identification of adjacency relations at the *n*-dimension levels. This is trivial on the direct map but is also true on the dual. Depending on the hierarchical level of a given cycle, different data exchange procedures or functions (in the programming sense) can be applied to compute different functions (in the ecological sense) of the element.

The lowest cycle is the β2 operation since β2 represents a local relationship between 2 components. This relationship may carry local interactions between these components: specific modeling and data sharing can be assessed at this level without the need to query information from the rest of the map

The orbitFace function defines a cycle bordering the element. This function can then carry the endogenic evolution of the element; it may include the evolution of embedded faces if there are any. In this context, modeling approaches can be defined on the full face and no specific data management has to be defined regarding other components of the system.

At the opposite, the orbitNode function defines a local cycle around all elements sharing a local adjacency. When previous function are mainly tools to move in maps, this function is of key interest for data sharing and exchange. Therefore, when modeling this aspect, it is necessary to use models of higher complexity, involving all the local interactions, but their relevance must be considered.

We can consider a similar analysis on the dual map. The dual graph explains which components interact mutually in the system and with the outside and which are considered as simply embedded. The major interest arises from the fact that, on the dual, the adjacencies are related to a global instead of a local scale, since faces (i.e., elements) are now considered as nodes. At the lower scale, a dart represents an oriented relation between 2 elements. The involution β2 applies to its inverse relation. Then, a dart indicates what comes out of the node β2 indicates what comes in the node. At the darts level, we can instantiate global information exchange/models in a nonsymmetric manner, since darts are oriented. The orbitFace now defines a cycle between the different nodes (elements) and defines then a framework to address the specific ecosystem services supported by these elements. The orbitNode of an element is also of interest. Indeed, we can consider global output and input in the agroforestry element, and their balance. In agroforestry context, it groups all input and output of an area.

**Combinatorial map transformation**

AFS and ecosystem services change over seasons and years in 2 possible ways:

• The structure of the system does not change, but the internal mechanisms evolve (i.e., ecosystem services such as microclimate buffering start to be produced as trees grow).
• The structure of the system is modified (addition or removal of plants, e.g., through tree thinning to keep only the best individuals after a few years of growth).

We can simulate the first type of change by modification on the attributes of darts. This operation is trivial in terms of code implementation and poses no risks of having unforeseeable repercussions on the system’s structure. The second corresponds to the addition (or deletion) of a node. It is an operation well known in combinatorial maps [37], respectively called sewing and cutting functions and defined on all the dimensions of the map simultaneously. Concretely, this operation updates all the darts and their respective permutation and involution.

**Fig. 6.**
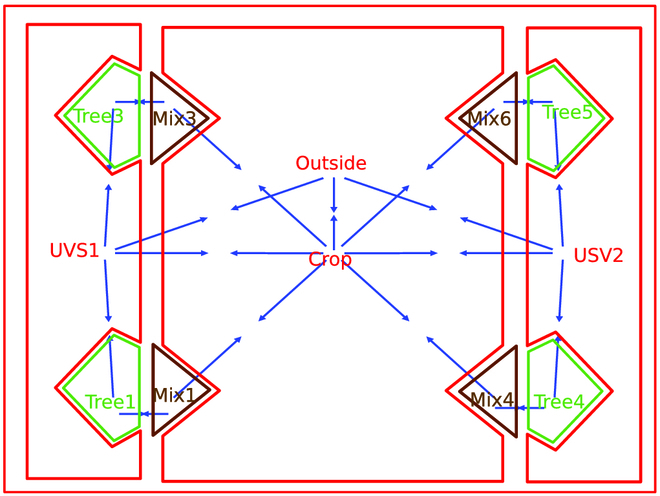
Combinatorial map (in red) and its dual (in blue) of the system represented in Fig. 1, several years later: the trees have grown and their canopy overlap the crop area. The grown trees areas are now split in 2 parts, on USV and on crop, on both graph and dual graph (in blue) generated by our application and postprocessed for improved readability.

Surprisingly, in the example presented in Application of combinatorial maps to AFS: A simple example, trees and crop are not connected. Tree–crop interaction can only be induce from through the strip component. Indeed, trees are still small in this case, and their representation in the strips is a small face. As trees grow and the projection of their crown overlaps with the cropped alley, the faces representing the trees will also expand and infringe on the crop face. Thus, the crop face subdivides into smaller faces corresponding to a mixing zone composed of crops overlapped by the canopy (in red in the Fig. 6). When updating the corresponding map, new local relationships will appear with dart adjacencies between the concerned trees borders and the crop border. Conversely, new global relationships will appear in the dual map between each concerned tree and the crop (in blue in Fig. 6).

### Higher-level operations: Pattern matching

Numerous operators that have been developed in graph theory can be very useful in an agroforestry context. We will detail here pattern matching.

**Fig. 7.**
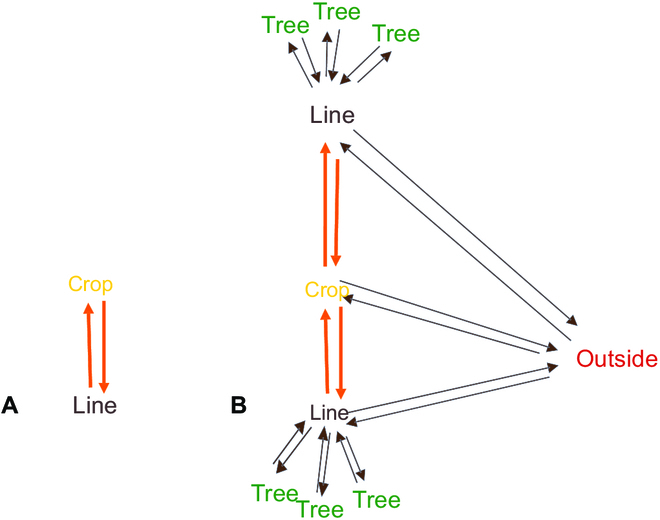
Example of pattern search. (A) A combinatorial map of ecosystem service that helps to regulate insect pest. (B) A combinatorial map of a plot that contains this ecosystem service twice.

During the agroforestry design process, actors could want to know if their system is already existing somewhere else or if a specific ecosystem service is present inside the system. To answer these questions, we must compare 2 (sub)systems, a task for which many pattern matching methods exist in graph theory [36], both for exact and approximate matching requests. In the example in Fig. 7, we search for the structure that supports a biotic interaction (e.g., regulation of pests by natural enemies) between an alley cropping and 2 tree lines serving as overwintering habitats for the natural enemies (Fig. 7A). In this case, we search exact matching between the substructure and the whole plot map. As can be seen in Fig. 7B, this pattern is present once. In addition, the pattern matching can use the darts’ information, so we can instantiate constraint-based attributes like “crop area must lay between two tree lines”. Note that the pattern matching can also apply to properties such as distances computed from faces’ coordinates; “the width of the alley cropping must be a multiple of the width of the farm cropping engine” is such a constraint.

### Implementation

Our implementation of the model (available at https://github.com/agroforestar/carte_combinatoire) is still under development. The prototype founds on 2 key ideas. First, our map is a 2D map based on 2D coordinates, which makes sense in the case of mapping the location of trees in an agricultural plot. Second, our implementation is incremental, meaning that the map is built by sequentially merging more and more complex faces. The system starts from a list of sets of coordinates describing the areas covered by the trees, the crops, and the understory vegetation strips, makes a face for each area, and then merges these individual faces until the whole system is included in the final map. The algorithms for creating and manipulating the maps come from the reference book by Damiand and Lienhardt [37], and we implemented them in Python 3.9. An important point to note is that all objects in the agroforestry system are considered as a face on our map even trees, which are usually considered as point objects.

We implemented most of the algorithms described by Damiand and Lienhardt [37]. In particular, we wrote the constructor (algorithm 22), the iterators (algorithms 28 to 30 and 34) and the functions to manipulate attributes of combinatorial map classes (algorithms 23 to 27). Then, we wrote the algorithms to create and remove darts (algorithms 35 and 36). We also implemented the algorithms to copy a map (algorithm 37) and to sew darts (algorithm 44). This set of methods allows to incrementally constructing the map from different faces representing the elements in the system.

**Fig. 8.**
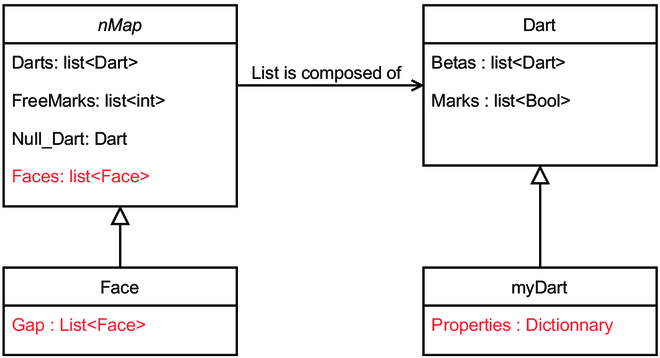
UML class diagram of our implementation of combinatorial map. In black, attribute already present in referenced book and, in red, attributes we add in our application.

In fact, this implementation was adapted to our context. We construct our class Face as a class inherited from class nMap (Fig. 8). Faces can have several holes. In case of a tree line face, a tree is representing by a hole in the strip face and filled with a face for the tree. We described the face class as an inheritance class from nMap (Fig. 8, left) with their holes. A hole is composed of darts and delimits a space filled with another face. The face definition can thus be considered as a recursive definition, and each level can be related to a specific scale. For instance, the area of a tree line can be considered as an entity at a given level, and its holes, corresponding to the individual tree areas, describe the area in more detail.

We introduce the Properties attribute in myDart, an inherited class of Dart (Fig. 8, right). It contains 2D coordinates, the type of the start node (tree, crop …) and may qualify other properties. We can simulate exchange between different areas into the plot and with the outside with the list of properties on darts.

## Discussion

### Advantages of the proposed framework compared to existing models

Despite the fact that the structure of AFS is of crucial importance to understand, predict, or optimize the production of target ecosystem services [26], to our knowledge, no agroforestry model used the concept of FSPM to represent the spatial organization at the system scale. In this article, we propose a new framework to describe AFS based on combinatorial maps.

This framework shows a good balance between the important criteria (complexity, capabilities to hold time, and space dynamics) for an efficient spatial representation targeting agroforestry system design (Table 1). Three features of the framework are of particular interest and show improvement over existing representations of AFS.

First, the 2 dual representations inherent to combinatorial maps allow representing not only the structure of the agroforestry system (as existing models already do) but also its functions. Indeed, our framework allows the user to focus alternatively on the areas, which represent the structure of the agroforestry system, and on the interfaces between areas, thus enabling to simulate the interactions between the elements of the system, which are the basis of ecosystem services. The model automatically ensures the consistency between both representations. This is consistent with the concept of FSPM, where the structure of a system (the plant) is directly linked to its functions. However, further research is needed to fully exploit this dual representation to build a complete Functional Structural Agroforestry Models approach.

Secondly, combinatorial maps allow a recursive description of the structure of the agroforestry system, so it becomes possible to describe the system in a hierarchical way. At the highest level, the system is described within an outside face, in which the different components are embedded. In the example presented above, at the second level, the system is composed of the areas of tree lines and of the crop. Each tree line area, in turn, embeds the different tree areas composing the tree line. However, the way the orbits are defined is the same and at all levels of the description. This authorizes a multiscale computation of ecosystem services, which is deemed particularly important to model complex AFS [41]. In particular, the fact that this framework can manage multiple scales allows aggregating individual plants at lower scales and adding a positive or negative impact of the system structure at higher scales. The combinatorial map and its dual define a framework that imposes that the interaction between the system elements must be modeled differently according to the local adjacencies and local relations. It also defines the cases where we need to mobilize models describing endogenous mechanisms or models describing interactions. For example, the fact that adjacencies between elements of the system are explicitly represented in the dual representation facilitates the simulation of local interactions between species. This recursive structure as well as the possibility of pattern matching is an asset for predicting ecosystem services either by reducing computation time (running relevant models’ implementation only on subsets of the whole agroforestry system) or by allowing to use a database of structure–service relationships.

The third advantage is that the consistency of the agroforestry model is enforced by the combinatorial map structure even when the system is modified. Thus, the description is robust and remains valid when adding or removing elements. Therefore, it can be used to design the representation of a new agroforestry system from scratch, as well as to modify an existing system represented either by geographical coordinates of plants or by a graph where nodes are the plants. This framework is also particularly well suited to represent the intrinsic dynamics of an agroforestry system, in which elements can appear (such as tree planting), disappear (such as tree thinning, or tree death), change their properties, or even impact the adjacencies as is the case when tree growth causes the tree canopy to project beyond the understory vegetation strip.

### Limits and perspectives

Although the proposed model fulfills all the criteria that we identified initially, some limits remain. For instance, global relations such as groups of nonadjacent elements (e.g., tree lines) are not represented directly in the combinatorial map. However, these relationships can be computing from the minimal topological graph [23]; this operation corresponds to an agroforestry pattern [26] extraction. Further work will allow full integration of the algorithms to do this into the prototype.

There is a need for further research in agroforestry, in particular to better understand and predict the link between species interactions and production of ecosystem services. Although this link has been highlighted for some time (e.g., [42]), work has only just started to automatize mining agronomical knowledge in relation with plant traits to predict the production of ecosystem services of different species associations (e.g., [43]), and the tree–service relationships are currently only able to relate services to single tree species, not species associations (e.g., [44]). Furthermore, to our knowledge, no trait–service database or tool take into account the spatial layout of associated species, despite the fact that its importance has been recognized [26]. Our framework could combine the notions of ecosystem service spatial unit and the relationship between species associations and biotic interactions in order to propose a modeling approach extending the principles of FSPM at the agroecosystem scale. Once we have built a catalog of patterns (i.e., tuples of agroforestry elements) that provide ecosystem services (potentially based on different biophysical models), our framework will allow us to analyze both structural and functional aspects of an agroforestry system.

Future applications of our framework will be useful to help farmers and advisors to design AFS and estimate their evolution through time. Integrating 2D or 3D visualization tools will facilitate the design process with a better communication [45] and help farmers to better project themselves and understand the impact of their choices on the system’s functioning. For example, we intend to use this framework to develop augmented reality tools that could be used either during agroforestry design workshops, for user-friendly, quick and robust, interactive ex ante evaluation of agroforestry system, or after the design phase, to visualize the future aspect of an agroforestry plot in the field.

### Conclusion

Modeling AFS is challenging due to the diversity of systems and the diversity of modeling objectives, from visualizing AFS during the design phase to running simulations to predict the functioning of the system. Our framework, inspired by FSPM, uses combinatorial maps to represent AFS. This framework allows focusing either on the agroforestry system structure (with the map representation) or on the system functioning (with its dual representation). The proposed approach ensures the consistency between both representations when the system evolves. Compared with existing representations of AFS, this framework combines the versatility of a graph-based representation with a spatial realism sufficient to represent irregularities in the pattern and the possibility to record geographical coordinates. Thus, our framework might serve as a unifying representation, with the possibility to export to and import from all other representations.

## Data Availability

The source code is available at https://github.com/agroforestar/carte_combinatoire.
